# Editorial: Social relationships and career development throughout the lifespan: identifying patterns of shared and non-shared agency

**DOI:** 10.3389/fpsyg.2023.1235829

**Published:** 2023-07-28

**Authors:** Esther S. Chang, Jacob Shane, Jonas Masdonati, Brandilynn J. Villarreal

**Affiliations:** ^1^Social and Behavioral Sciences, Soka University of America, Aliso Viejo, CA, United States; ^2^City College of New York (CUNY), New York City, NY, United States; ^3^Research Center in Vocational Psychology and Career Counseling, Institute of Psychology, University of Lausanne, Lausanne, Vaud, Switzerland; ^4^Cal Poly Humboldt, Arcata, CA, United States

**Keywords:** career development, career goals, career socialization, career social support, lifespan development, lifespan control

This Research Topic aimed to identify how close social relationships can benefit or impede career development through the lifespan. The Research Topic includes examinations of both early and mid-career adults to better speak to the distinct challenges and opportunities that emerge during different phases of career development. For example, adolescents and young adults must prepare for and establish their careers as the world's economies grow more integrated and interdependent, leading them to navigate complex and unpredictable career possibilities and socio-cultural standards for success. Middle-aged and older adults must balance the competing demands of work, health, and family, and may be more vulnerable to unexpected career changes such as those brought about by technological innovations and the COVID-19 pandemic. Although formal institutional and professional assistance and guidance is important for career development, many adults may lack the connections, time, or knowledge to access or fully make use of these potential resources. Instead, adults may turn to their close social relationships beyond the workplace to scaffold their career development as they choose, pursue, and disengage from their career goals.

In this Research Topic, we include studies of parents (Dmitrieva and Espel; LeBlanc and Lyons) including how young men's identity as a father anchors their career identity (Crafford and Koekemoer), the relative influence of different relationship partners during young adulthood (Chang et al.), the types and sources of social support that are available for people undergoing career transitions (Greer and Kirk; Masdonati et al.), and the sacrifices that people make in their own career development to improve the quality of their close social relationships (Zikic). The papers in this Research Topic use different lenses with different focal points, yet converge on the need to understand the web of social relationships that structure, support, or strangle an individual's career development.

Greer and Kirk's close look at women's social networks allows for an appreciation of the twists and turns that are involved in careers because of family and caregiving responsibilities. Although the authors identify at least eight sources of support during career transitions, they find individuals must actively seek this support. Thus, women who are more likely to have successful career transitions must be engaged in both their career goals and in building and maintaining an involved network of family and friends to support these goals.

As explained in Zikic concept paper, conflicts between career and social relationships may lead to career sacrifice wherein one's career goals are changed or set aside. Work is only one of many life domains, and letting go of some work-related goals may help further development in other domains, such as family relationships that provide purpose and happiness. Recent changes, such as the rise of remote work, may lead people to sacrifice advancement-type career goals for the ability to spend more time with friends and family. In this, and other ways, career sacrifice may become more common, more prosocial, and less regretful.

The empirical research included in this Research Topic uses qualitative or quantitative methods on adults from different settings and different regions around the world. The studies included here give voice to individuals struggling to achieve career goals across a wide range of settings, from adjudicated adolescent females in the United States (Dmitrieva and Espel) to highly educated young fathers in dual-earner relationships in South Africa (Crafford and Koekemoer). In addition, studies examine how young adults in Canada (LeBlanc and Lyons) and the United States (Chang et al.; Dmitrieva and Espel) prepare and launch their careers, and how middle-aged adults cope with involuntary career changes in Europe (Masdonati et al.). A common theme across these studies is that one's career goals can be viewed as social projects. When pursued jointly, these social projects can be for the better (Chang et al.; Crafford and Koekemoer) but when they are pursued without others, they can be for the worse (Dmitrieva and Espel; LeBlanc and Lyons; Masdonati et al.).

The collective results from studies of people transitioning into adulthood suggest that social networks can provide an important developmental resource. These studies also speak to the unique roles that mothers and fathers play, and the need to consider both sources of influence separately when examining parental influences on career development (Chang et al.; Dmitrieva and Espel; LeBlanc and Lyons). As parenting extends into the adult years, these studies lend support to parenting practices that provide warmth (Dmitreiva and Espel), and respect their child's autonomy (LeBlanc and Lyons) and agency (Chang et al.). However, parental influences may wane as children reach later stages of young adulthood and turn to their own romantic partners and find other important non-parental adults to support, guide, and inform their career development (Chang et al.).

Two qualitative studies of young and middle-aged adults in this Research Topic reveal that middle adulthood may no longer be a time to accommodate to others. As seen in dual-earner couples in South Africa, families must cultivate a “deliberate life” to regulate two career goals and their family needs (Crafford and Koekemoer). Although career decisions are often socialized as individual choices, the reality for many is that the arrival of children into the family creates an observable shift in priorities. The fathers in Crafford and Koekemoer show us that careers can be molded to accommodate parenting values and parenting styles as long as there is planning and negotiation with spouses.

As important as our informal social networks can be for career development, it is critical to recognize that family and friends are not always equipped to help individuals cope with involuntary career changes (i.e., layoffs, demotions, etc.). Masdonati et al. described how participants experiencing involuntary career changes felt shame and that they were a burden to others. Their participants also discuss how the institutional support of professional counselors, career counseling programs, and financial support via disability or unemployment insurance was critical in giving them time to reflect on their career change and start working again. For those who struggle with career entry (such as adjudicated older youth, Dmitreiva and Espel) or with an overwhelming loss of control over career direction, formal support may not be replaced by informal relationships with others.

Collectively, this Research Topic examines the extent to which careers are social projects that can be accomplished through joint efforts with others (shared agency) or become unrealized because joint effort was not actualized (non-shared agency). This collection of articles shows that patterns of career shared and non-shared agency exist with family, friends, mentors, and romantic partners. Future research should seek to understand how these shared and non-shared agency patterns co-develop with one's own career development, including the specific roles that different social partners play and how these influences wax and wane as individuals progress throughout their lifespan.

## Author contributions

EC wrote the initial draft of the editorial, contributed substantially to the conceptualization and development of the ideas presented, and took the lead in organizing and structuring the content, ensuring its coherence and clarity. JS played a crucial role in the editing and revision process of the manuscript, provided valuable feedback, critically reviewed the content, and contributed to refining the language, style, and overall presentation of the work. JM and BV have made significant contributions to the overall project and participated in the editing of the special issue and this editorial. All authors reviewed and approved the final version of the editorial before submission.

